# Characteristics and Outcomes Among Adults Aged ≥60 Years Hospitalized with Laboratory-Confirmed Respiratory Syncytial Virus — RSV-NET, 12 States, July 2022–June 2023

**DOI:** 10.15585/mmwr.mm7240a1

**Published:** 2023-10-06

**Authors:** Fiona P. Havers, Michael Whitaker, Michael Melgar, Bhoomija Chatwani, Shua J. Chai, Nisha B. Alden, James Meek, Kyle P. Openo, Patricia A. Ryan, Sue Kim, Ruth Lynfield, Yomei P. Shaw, Grant Barney, Brenda L. Tesini, Melissa Sutton, H. Keipp Talbot, Kristen P. Olsen, Monica E. Patton, RSV-NET Surveillance Team, RSV-NET Surveillance Team, Pam Daily Kirley, Elizabeth Austin, Daewi Kim, Chandler Surell, Maya Monroe, Lauren Leegwater, Erica Mumm, Molly Bleecker, Adam Rowe, Kevin Popham, Arilene Novak, William Schaffner, Holly Staten

**Affiliations:** ^1^Coronavirus and Other Respiratory Viruses Division, National Center for Immunization and Respiratory Diseases, CDC; ^2^Eagle Health Analytics, LLC., Atlanta, Georgia; ^3^California Emerging Infections Program, Oakland, California; ^4^Career Epidemiology Field Officer Program, CDC; ^5^Colorado Department of Public Health & Environment; ^6^Connecticut Emerging Infections Program, Yale School of Public Health, New Haven, Connecticut; ^7^Emory University School of Medicine, Atlanta, Georgia; ^8^Georgia Emerging Infections Program, Georgia Department of Public Health; ^9^Atlanta Veterans Affairs Medical Center, Decatur, Georgia; ^10^Maryland Department of Health; ^11^Michigan Department of Health & Human Services; ^12^Minnesota Department of Health; ^13^New Mexico Department of Health; ^14^New York State Department of Health; ^15^University of Rochester School of Medicine and Dentistry, Rochester, New York; ^16^Public Health Division, Oregon Health Authority; ^17^Vanderbilt University Medical Center, Nashville, Tennessee; ^18^Salt Lake County Health Department, Salt Lake City, Utah.; California Emerging Infections Program; Colorado Department of Public Health & Environment; Connecticut Emerging Infections Program, Yale School of Public Health; Emory University School of Medicine, Georgia Emerging Infections Program, Georgia Department of Public Health, Atlanta Veterans Affairs Medical Center; Maryland Department of Health; Michigan Department of Health & Human Services; Minnesota Department of Health; University of New Mexico Emerging Infections Program; New York State Department of Health; University of Rochester School of Medicine and Dentistry; Public Health Division, Oregon Health Authority; Vanderbilt University Medical Center; Salt Lake County Health Department.

**Summary**

**What is already known about this topic?**

Respiratory syncytial virus (RSV) causes substantial morbidity and mortality in older adults. In June 2023, CDC recommended RSV vaccination for adults aged ≥60 years, using shared clinical decision-making and prioritizing those at highest risk for severe disease.

**What is added by this report?**

Among 1,634 patients aged ≥60 years hospitalized with RSV, 54% were aged ≥75 years, and 17% resided in long-term care facilities (LTCFs). Obesity, chronic obstructive pulmonary disease (COPD), and congestive heart failure (CHF) were common underlying conditions.

**What are the implications for public health practice?**

Clinicians and patients should consider age (particularly age ≥75 years), LTCF residence, and underlying medical conditions, including COPD and CHF, in shared decision-making regarding RSV vaccination to prevent severe RSV-associated outcomes.

## Abstract

Respiratory syncytial virus (RSV) causes substantial morbidity and mortality in older adults. In May 2023, two RSV vaccines were approved for prevention of RSV lower respiratory tract disease in adults aged ≥60 years. In June 2023, CDC recommended RSV vaccination for adults aged ≥60 years, using shared clinical decision-making. Using data from the Respiratory Syncytial Virus–Associated Hospitalization Surveillance Network, a population-based hospitalization surveillance system operating in 12 states, this analysis examined characteristics (including age, underlying medical conditions, and clinical outcomes) of 3,218 adults aged ≥60 years who were hospitalized with laboratory-confirmed RSV infection during July 2022–June 2023. Among a random sample of 1,634 older adult patients with RSV-associated hospitalization, 54.1% were aged ≥75 years, and the most common underlying medical conditions were obesity, chronic obstructive pulmonary disease, congestive heart failure, and diabetes. Severe outcomes occurred in 18.5% (95% CI = 15.9%–21.2%) of hospitalized patients aged ≥60 years. Overall, 17.0% (95% CI = 14.5%–19.7%) of patients with RSV infection were admitted to an intensive care unit, 4.8% (95% CI = 3.5%–6.3%) required mechanical ventilation, and 4.7% (95% CI = 3.6%–6.1%) died; 17.2% (95% CI = 14.9%–19.8%) of all cases occurred in long-term care facility residents. These data highlight the importance of prioritizing those at highest risk for severe RSV disease and suggest that clinicians and patients consider age (particularly age ≥75 years), long-term care facility residence, and underlying medical conditions, including chronic obstructive pulmonary disease and congestive heart failure, in shared clinical decision-making when offering RSV vaccine to adults aged ≥60 years.

## Introduction

Respiratory syncytial virus (RSV) causes substantial morbidity and mortality in older adults, resulting in approximately 60,000–160,000 hospitalizations and 6,000–10,000 deaths annually among adults aged ≥65 years (*1*). In May 2023, the Food and Drug Administration approved two RSV vaccines for prevention of RSV lower respiratory tract disease in adults aged ≥60 years.* In June 2023, CDC recommended RSV vaccination for adults aged ≥60 years using shared clinical decision-making between patient and clinicians;^†^ adults at highest risk for severe RSV disease are most likely to benefit and should be prioritized for vaccination (*1*). To describe persons who experienced severe RSV disease requiring hospitalization, data from a large, geographically diverse surveillance system were analyzed to identify characteristics of adults aged ≥60 years hospitalized with laboratory-confirmed RSV infection during the 2022–23 respiratory virus season.

## Methods

The Respiratory Syncytial Virus–Associated Hospitalization Surveillance Network (RSV-NET)^§^ conducts population-based surveillance for RSV-associated hospitalizations in approximately 300 hospitals in 58 counties across 12 states,^¶^ covering approximately 9% of the U.S. population. RSV-NET identifies residents within the network catchment area who are hospitalized with positive RSV tests results for provider-ordered reverse transcription–polymerase chain reaction (RT-PCR) or rapid antigen detection tests during their hospitalization or during the 14 days preceding admission.

Because the 2022–23 RSV season started earlier than did seasons preceding the COVID-19 pandemic (*2*), this description of demographic characteristics of hospitalized RSV-NET patients includes those hospitalized during July 1, 2022–June 30, 2023. Using previously described methods (*3*), clinical data were collected by trained surveillance officers from a random sample of medical charts for adults hospitalized during October 1, 2022–April 30, 2023, and stratified by age and site. Sampled data are presented as unweighted case counts and weighted percentages that were weighted for the probability of selection and adjusted to better represent the hospitalized population of the catchment area (*3*). Age distributions of patients aged ≥60 years who were hospitalized and experienced severe outcomes, defined as intensive care unit (ICU) admission, mechanical ventilation, and in-hospital death, were compared with the overall age distribution of persons ≥60 years in the RSV-NET catchment area. Underlying medical conditions among hospitalized patients and those with severe outcomes were assessed and described. Data were analyzed using SAS survey procedures (version 9.4; SAS Institute). Differences were assessed using chi-square tests; p-values <0.05 were considered statistically significant. This activity was reviewed by CDC, deemed not research, and was conducted consistent with applicable federal law and CDC policy.**

## Results

Among 3,218 adults aged ≥60 years with an identified RSV-associated hospitalization during July 2022–June 2023, a total of 1,738 (54.0%) were aged ≥75 years (this group constituted 29.0% of the catchment population of adults aged ≥60 years); 434 (13.5%) and 1,208 (37.5%) of RSV-associated hospitalizations occurred in persons aged 60–64 and ≥80 years, respectively. Overall, 222 (6.9%) patients were Hispanic or Latino (Hispanic), 2,159 (67.2%) patients were non-Hispanic White (White), 496 (15.4%) non-Hispanic Black or African American (Black), 228 (7.1%) non-Hispanic Asian or Pacific Islander (A/PI), 13 (0.4%) non-Hispanic American Indian or Alaska Native (AI/AN) persons, and 100 (3.2%) persons were of other or unknown race. The median patient age was 75 years (IQR = 68–84 years). The median age of White patients (77 years; IQR = 69–85 years) was significantly higher than that of patients who were Black (70 years; IQR = 65–77), Hispanic (74 years; IQR = 66–83 years), or AI/AN (72 years; IQR = 71–75 years) and was lower than that among A/PI (79 years; IQR = 71–87 years) patients (p-value <0.01 for all) (Supplementary Table 1; https://stacks.cdc.gov/view/cdc/133296). The proportion of hospitalized patients whose race was reported as Hispanic or Black decreased with increasing age (p-value <0.01); Black patients accounted for 28.2% of hospitalized patients aged 60–64 years and 8.2% of those aged ≥80 years (Supplementary Table 2; https://stacks.cdc.gov/view/cdc/133297).

Among a random sample of 1,634 adults aged ≥60 years hospitalized during October 2022–April 2023 whose medical charts were reviewed, 54.1% were aged ≥75 years, and 290 (17.2%) were long-term care facility (LTCF) residents, including 175 (26.9%) of those aged ≥80 years (Table). Nearly all patients (1,553 [95%]) had SARS-CoV-2 test results available, among which 39 (2.4%) were positive; 1,587 (97.1%) had influenza testing results, among which 23 (2.2%) were positive.^††^ Prevalence of severe outcomes was not higher among patients with viral codetections compared with those with RSV alone detected (p>0.5). The median length of hospitalization was 4.1 days (IQR = 2.2–7.6 days). A substantial proportion (332 [18.5%; 95% CI = 15.9%–21.2%]) of patients had at least one severe outcome, including 297 (17.0%) who required ICU admission, 94 (4.8%) who required mechanical ventilation, and 98 (4.7%) who died while hospitalized.

**TABLE:** Characteristics of a random sample of patients aged ≥60 years hospitalized with laboratory-confirmed respiratory syncytial virus infection* (N = 1,634), stratified by age and site — Respiratory Syncytial Virus–Associated Hospitalization Surveillance Network, 12 states,**^†^** October 2022–April 2023

Characteristic	Age group, yrs							
	Overall		60–69		70–79		≥80	
	No.	Weighted % (95% CI)	No.	Weighted % (95% CI)	No.	Weighted % (95% CI)	No.	Weighted % (95% CI)
**Total, row %**	**1,634**	**100**	**523**	**32**	**554**	**34**	**557**	**34**
**Sex**								
Female	975	60.5 (57.0–63.8)	311	60.7 (54.8–66.4)	317	57.5 (51.7–63.1)	347	62.8 (56.7–68.7)
Male	659	39.5 (36.2–43.0)	212	39.3 (33.6–45.2)	237	42.5 (36.9–48.3)	210	37.2 (31.3–43.3)
**Race and ethnicity** ^§^								
AI/AN	7	0.3 (0.1–0.7)	3	0.5 (0.1–1.5)	4	0.5 (0.1–1.5)	0	—
A/PI, NH	95	7.1 (5.2–9.5)	31	7.3 (3.6–12.8)	23	3.9 (2.3–6.2)	41	9.8 (6.1–14.6)
Black or African American, NH	213	13.0 (11.0–15.2)	111	22.4 (18.0–27.4)	69	13.0 (9.6–17.0)	33	5.7 (3.5–8.7)
White, NH	1,181	70.2 (67.0–73.3)	333	60.6 (54.6–66.4)	404	70.2 (64.7–75.4)	444	77.6 (72.1–82.4)
Hispanic or Latino	92	6.7 (5.0–8.7)	33	7.2 (4.4–11.0)	33	9.1 (5.5–13.9)	26	4.2 (2.4–6.7)
All other races^¶^	5	0.4 (0.1–1.3)	1	0.1 (0.0–0.9)	2	0.3 (0.0–1.2)	2	0.7 (0.0–3.3)
Unknown	41	2.3 (1.6–3.3	11	1.9 (0.8–3.6)	19	3.0 (1.7–4.9)	11	2.0 (0.9–3.9)
LTCF residence**	290	17.2 (14.9–19.8)	36	5.8 (3.8–8.5)	79	16.1 (12.0–20.9)	175	26.9 (22.2–32.0)
**Viral codetection** ^††^								
SARS-CoV-2	39	2.4 (1.5–3.6)	11	1.6 (0.7–3.1)	19	3.4 (1.7–5.9)	9	2.2 (0.8–4.9)
Influenza	23	2.2 (1.2–3.8)	7	1.9 (0.4–5.0)	9	2.3 (0.6–5.7)	7	2.4 (0.8–5.5)
**Hospitalization outcome^§§^**								
Hospital stay, days, median (IQR)	4.1 (2.2–7.6)	—	4.0 (2.0–7.4)	—	4.1 (2.3–7.7)	—	4.2 (2.2–7.7)	—
BiPAP/CPAP	339	19.8 (17.3–22.6)	116	23.3 (18.3–28.9)	131	22.6 (18.1–27.6)	92	14.8 (11.2–19.2)
High-flow nasal cannula	80	4.3 (3.2–5.7)	22	3.9 (2.1–6.7)	31	5.4 (3.3–8.2)	27	3.7 (2.2–5.8)
≥1 severe outcome^¶¶^	332	18.5 (15.9–21.2)	112	20.5 (16.3–25.3)	124	22.3 (17.2–28.1)	96	13.7 (10.2–17.8)
ICU admission	297	17.0 (14.5–19.7)	111	20.5 (16.2–25.2)	110	20.6 (15.5–26.4)	76	11.3 (8.0–15.4)
Invasive mechanical ventilation	94	4.8 (3.5–6.3)	42	6.4 (4.4–9.0)	33	4.9 (2.9–7.7)	19	3.5 (1.4–6.9)
In-hospital death	98	4.7 (3.6–6.1)	22	3.0 (1.7–4.8)	39	5.8 (3.7–8.5)	37	5.2 (3.2–7.9)
**Underlying medical condition**								
≥1 underlying medical condition***	1,584	95.5 (93.2–97.2)	501	96.3 (94.0–97.9)	540	97.2 (95.1–98.6)	543	93.5 (87.3–97.2)
Chronic lung disease	813	49.2 (45.7–52.7)	290	54.4 (48.2–60.4)	292	53.9 (48.0–59.7)	231	41.2 (35.3–47.3)
COPD	552	33.7 (30.5–37.0)	197	38.9 (33.1–44.8)	189	34.4 (28.9–40.4)	166	29.1 (24.0–34.6)
Asthma	332	19.1 (16.6–21.8)	134	25.4 (20.4–31.0)	108	16.5 (12.9–20.7)	90	16.4 (12.3–21.2)
Other^†††^	72	5.4 (3.8–7.3)	17	3.0 (1.6–5.1)	34	8.4 (5.0–13.1)	21	4.6 (2.4–8.0)
Cardiovascular disease	1,108	67.1 (63.7–70.5)	304	55.0 (48.8–61.0)	371	67.5 (61.8–72.8)	433	76.3 (70.0–81.8)
CHF^§§§^	545	33.2 (30.0–36.5)	165	31.5 (26.1–37.2)	165	29.8 (24.4–35.7)	215	37.4 (31.7–43.4)
CAD^¶¶¶^	435	26.4 (23.5–29.5)	109	20.9 (16.3–26.3)	151	28.8 (23.7–34.4)	175	28.6 (23.6–34.1)
CVA****	253	13.7 (11.7–15.9)	55	9.6 (6.9–13.0)	90	14.0 (10.7–17.8)	108	16.7 (12.8–21.1)
Immunocompromising condition	292	18.6 (16.0–21.4)	101	19.0 (14.5–24.1)	121	22.8 (18.0–28.1)	70	14.8 (10.8–19.6)
Diabetes mellitus	553	32.6 (29.5–35.8)	200	38.0 (32.4–43.9)	195	32.7 (27.6–38.1)	158	28.4 (23.1–34.2)
Neurologic condition	439	27.3 (24.3–30.5)	96	17.3 (13.4–21.7)	135	25.2 (20.3–30.6)	208	36.8 (31.0–42.9)
Dementia^††††^	183	12.4 (10.1–15.0)	7	1.0 (0.4–2.4)	40	8.5 (5.5–12.5)	136	24.5 (19.4–30.1)
Other	256	14.9 (12.6–17.4)	89	16.2 (12.5–20.6)	95	16.7 (12.6–21.4)	72	12.3 (8.8–16.6)
Kidney disorder	477	29.3 (26.3–32.5)	134	24.7 (19.7–30.1)	156	30.0 (24.8–35.5)	187	32.3 (26.9–38.0)
Obesity	572	37.8 (34.3–41.4)	230	46.4 (40.3–52.5)	213	42.4 (36.5–48.6)	129	27.1 (21.3–33.6)

**Abbreviations:** AI/AN = American Indian or Alaska Native; A/PI = Asian or other Pacific Islander; BiPAP/CPAP = bilevel positive airway pressure/continuous positive airway pressure; CAD = coronary artery disease; CHF = congestive heart failure; COPD = chronic obstructive pulmonary disease; CVA = cerebrovascular accident; ICU = intensive care unit; LTCF = long-term care facility; NH = non-Hispanic.

* Data are from a weighted sample of hospitalized adults with completed medical record abstractions. Sample sizes presented are unweighted with weighted percentages.

^†^ Includes persons admitted to a hospital with an admission date during October 1, 2022–April 30, 2023. Selected counties in California, Colorado, Connecticut, Georgia, Maryland, Michigan, Minnesota, New Mexico, New York, Oregon, Tennessee, and Utah.

^§^ If ethnicity was unknown, NH ethnicity was assumed.

^¶^ Includes NH persons reported as other or multiple races.

** LTCF residents include hospitalized adults who were identified as residents of a nursing home or skilled nursing facility, rehabilitation facility, assisted living or residential care, long-term acute care hospital, group or retirement home, or other LTCF upon hospital admission. A free-text field for other types of residences was examined; patients with an LTCF-type residence were also categorized as LTCF residents.

^††^ Results reported among adults who received testing (as opposed to all hospitalized adults). Because of testing practices, denominators differed among the viral respiratory pathogens based on type of test results available: SARS-CoV-2 = 95% (1,553) and influenza (influenza A, influenza B, and flu [not subtyped]) = 97% (1,587). Among 375 (24.2%) patients who received testing for other viruses, 15 additional viruses were detected: nine rhinoviruses, four seasonal coronaviruses, and two parainfluenza viruses.

^§§^ Hospitalization outcomes are not mutually exclusive categories, and patients can be included in more than one category.

^¶¶^ Severe outcome is defined as requiring ICU admission or mechanical ventilation or experiencing in-hospital death.

*** Defined as one or more of the following: chronic lung disease, including asthma; chronic metabolic disease including diabetes mellitus; blood disorder or hemoglobinopathy; cardiovascular disease; neurologic disorder; immunocompromising condition; renal disease; gastrointestinal or liver disease; rheumatologic, autoimmune, or inflammatory condition; obesity; feeding tube dependency; and wheelchair dependency.

^†††^ Other chronic lung diseases include interstitial lung disease, pulmonary fibrosis, restrictive lung disease, sarcoidosis, asbestosis, and chronic respiratory failure including oxygen dependence.

^§§§^ CHF includes cardiomyopathy, heart failure with preserved ejection fraction, and heart failure with reduced ejection fraction.

^¶¶¶^ CAD includes history of coronary artery bypass graft and myocardial infarction.

**** CVA includes history of stroke or transient ischemic attack.

^††††^ Dementia includes Alzheimer disease and other types of dementia.

**FIGURE 1:**
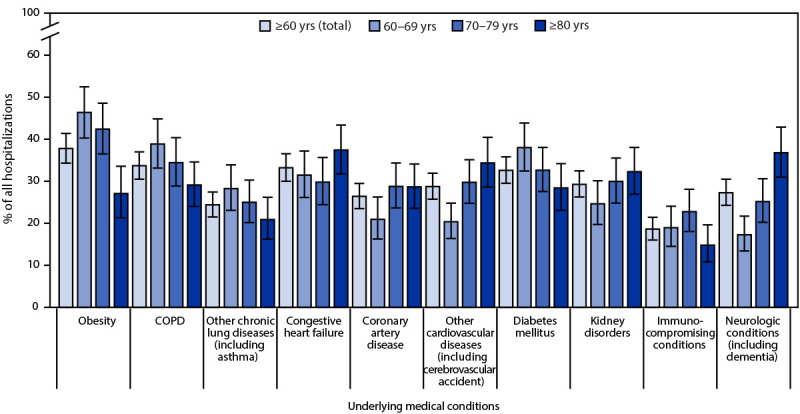
Underlying medical conditions*^,^^†^ among patients hospitalized with laboratory-confirmed respiratory syncytial virus infection^§^ — Respiratory Syncytial Virus–Associated Hospitalization Surveillance Network, 12 states,^¶^ October 2022–April 2023 **Abbreviation:** COPD = chronic obstructive pulmonary disease. * With 95% CIs indicated by error bars. ^†^ Congestive heart failure includes cardiomyopathy; coronary artery disease includes history of coronary artery bypass graft and myocardial infarction; cerebrovascular accident includes history of stroke or transient ischemic attack; dementia includes Alzheimer disease and other types of dementia. ^§^ Data are from a weighted sample of hospitalized adults with completed medical record abstractions. Sample sizes presented are unweighted with weighted percentages. ^¶^ Select counties in California, Colorado, Connecticut, Georgia, Maryland, Michigan, Minnesota, New Mexico, New York, Oregon, Tennessee, and Utah.

Almost all sampled patients (1,584; 95.5%) had at least one underlying medical condition, most commonly obesity (37.8%), chronic obstructive pulmonary disease (COPD) (33.7%), congestive heart failure (CHF) (33.2%), and diabetes mellitus (32.6%); 18.6% had an immunocompromising condition (Table) (Figure 1). The following underlying conditions were significantly more prevalent in patients with severe outcomes than in those without severe outcomes: COPD (40.0% versus 32.0%; p = 0.047), other chronic lung diseases excluding COPD and asthma (9.1% versus 4.4%; p = 0.04), and CHF (41.2% versus 31.4%; p = 0.01).

**FIGURE 2:**
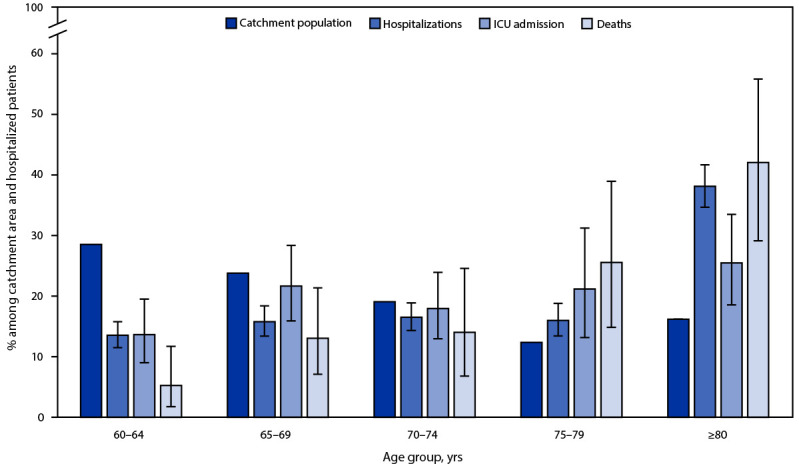
Age distribution* among persons aged ≥60 years residing in the surveillance network catchment area^†^ and among laboratory-confirmed respiratory syncytial virus–associated hospitalizations, intensive care unit admissions, and in-hospital deaths — Respiratory Syncytial Virus–Associated Hospitalization Surveillance Network, 12 states, October 2022–April 2023 **Abbreviations:** ICU = intensive care unit; RSV = respiratory syncytial virus; RSV-NET = Respiratory Syncytial Virus–Associated Hospitalization Surveillance Network. * With 95% CIs indicated by error bars. ^†^ The RSV catchment area includes select counties in California, Colorado, Connecticut, Georgia, Maryland, Michigan, Minnesota, New Mexico, New York, Oregon, Tennessee, and Utah. RSV-associated hospitalizations among RSV-NET catchment area residents have hospital admission dates from October 1, 2022 through April 30, 2023. Those with severe RSV disease might be more likely to receive RSV testing; therefore, these data could potentially overestimate the proportion of severe outcomes among hospitalized patients.

Whereas adults aged 75–79 years and ≥80 years accounted for 12.4% and 16.2% of the catchment area populations, respectively (Figure 2), they accounted for 16.0% (95% CI = 13.5%–18.8%) and 38.1% (95% CI = 34.7%–41.7%) of hospitalizations, 21.2% (95% CI = 13.2%–31.3%) and 25.5% (95% CI = 18.6%–33.5%) of ICU admissions, and 25.6% (95% CI = 14.8%–39%) and 42.1% (95% CI = 29.1%–55.9%) of in-hospital deaths, respectively. Orders to not resuscitate or intubate were in place for 321 (20%) patients, including 211 (35%) patients aged ≥80 years.

## Discussion

During July 2022–June 2023, RSV-associated hospitalizations among adults aged ≥60 years in a large population-based surveillance system occurred predominantly among those aged ≥75 years (54%); many (17.2%) of these patients resided in long-term care facilities. The median age of hospitalized AI/AN, Black, and Hispanic patients was lower than that of hospitalized White patients. Viral coinfections reported in RSV-NET were infrequent, despite comprehensive testing for SARS-CoV-2 and influenza, indicating that RSV alone caused substantial morbidity and mortality in this population. Most patients hospitalized with RSV had underlying medical conditions, notably CHF and COPD, which were associated with severe outcomes. Severe outcomes were common, with 17.0% of hospitalized patients requiring ICU admission and nearly 5% dying during their hospitalization.

CDC recommends RSV vaccination for adults aged ≥60 years using shared clinical decision-making, which may consider a patient’s individual risk for severe disease (*1*). Adults aged ≥75 years were overrepresented among older adult RSV-NET hospitalizations, consistent with previous studies demonstrating increased RSV hospitalization rates with increasing age (*4*,*5*). However, the median age of hospitalized older adults who were AI/AN, Black, and Hispanic patients was lower than that for White patients, such that persons in these three groups accounted for a larger proportion of RSV-NET hospitalizations among the younger age groups. This finding likely reflects different age distributions, as well as life expectancy, within the catchment population, as well as potentially higher risk for hospitalization at younger ages resulting from racial and ethnic disparities in underlying medical conditions, access to medical care, and socioeconomic status (*6*–*8*).

The prevalence of underlying medical conditions among hospitalized patients was high, including CHF and COPD, both of which were disproportionately associated with severe outcomes in this analysis. Both CHF and COPD have been previously associated with increased RSV hospitalization rates (*4*,*5*). One study indicated that older adults with COPD (aged ≥65 years) and CHF (aged 60–79 years) had RSV hospitalization rates that were 3.5–13.4 times and 5.9–7.6 times higher, respectively, than rates among those without those conditions (*5*). The large proportion of LTCF residents among RSV-NET hospitalizations is also consistent with published literature demonstrating this population’s vulnerability to institutional outbreaks and hospitalization (*9*).

### Limitations

The findings in this report are subject to at least three limitations. First, RSV-associated hospitalizations might have been missed because of test availability or clinician testing practices that limit RSV testing among hospitalized adults. Second, and conversely, severely ill patients might have been more likely to undergo RSV testing, potentially overestimating the proportion of severe outcomes among hospitalized patients. Finally, because RSV-NET covers 9% of the U.S. population, these findings might not be nationally generalizable.

### Implications for Public Health Practice

RSV causes substantial morbidity and mortality in adults aged ≥60 years; these findings suggest that advanced age (particularly ≥75 years), LTCF residence, and the presence of underlying medical conditions, including COPD and CHF, might be risk factors for clinicians and patients to consider in shared decision-making regarding RSV vaccination. It is important that special attention be paid to equitable access to vaccines for AI/AN, Black, and Hispanic adults, who were hospitalized for RSV at younger ages than were White adults.

## Footnotes

* GlaxoSmithKline vaccine: https://www.fda.gov/media/167806/download; Pfizer vaccine: https://www.fda.gov/media/168890/download

† Unlike age- and risk-based recommendations, for which the default decision should be to vaccinate the patient unless vaccination is contraindicated, shared clinical decision-making recommendations have no default. The decision of whether to vaccinate may consider the best available evidence regarding who would benefit from vaccination; the individual patient’s characteristics, values, and preferences; the vaccine characteristics; and the clinician’s discretion. https://www.cdc.gov/vaccines/acip/acip-scdm-faqs.html

§ RSV-NET is one of three Respiratory Virus Hospitalization Surveillance Network (RESP-NET) platforms that conduct population-based surveillance for hospitalizations for RSV (RSV-NET), COVID-19 (COVID-NET), and influenza (FluSurv-NET). https://www.cdc.gov/surveillance/resp-net/dashboard.html

¶ Selected counties in California, Colorado, Connecticut, Georgia, Maryland, Michigan, Minnesota, New Mexico, New York, Oregon, Tennessee, and Utah. https://www.cdc.gov/rsv/research/rsv-net/overview-methods.html

** 45 C.F.R. part 46.102(l)(2), 21 C.F.R. part 56; 42 U.S.C. Sect. 241(d); 5 U.S.C. Sect. 552a; 44 U.S.C. Sect. 3501 et seq.

†† Among 375 (24.2%) patients receiving testing for other viruses, nine rhinovirus, four seasonal coronavirus, and two parainfluenza virus codetections were identified.
